# Is It Time to Adopt the LM‐View Fluoroscopic Projection in Balloon‐Expandable TAVR?

**DOI:** 10.1002/ccd.70552

**Published:** 2026-03-09

**Authors:** Julius Jelisejevas, John G. Webb

**Affiliations:** ^1^ Centre for Cardiovascular Innovation/Center for Heart Valve Innovation, St Paul's and Vancouver General Hospital Vancouver Canada; ^2^ Department of Cardiology University and Hospital of Fribourg Fribourg Switzerland; ^3^ Cardiovascular Translational Laboratory, Providence Research & Centre for Heart Lung Innovation Vancouver Canada

**Keywords:** index‐TAVR, LM‐View, redo‐TAVR

Balloon‐expandable transcatheter heart valves (THV) are typically deployed in a three‐cusp (3C) coplanar projection, with selective use of complementary cusp‐overlap (CO) view to optimize implantation depth and reduce risk of conduction disturbances [1]. Although techniques aimed at minimizing pacemaker risk may be prioritized in older patients, the expansion of transcatheter aortic valve replacement (TAVR) into younger populations underscores the importance of recognizing the spatial relationship between the THV stent‐frame outflow (neo‐skirt plane) and the left main (LM) ostium (coronary risk plane). Failure to do so may compromise future redo strategies should patients outlive their index THV, as shallower implantation has been associated with reduced redo‐TAVR feasibility [2]. Having recently introduced the LM‐View implantation technique as a complementary implantation projection visualizing LM ostium during TAVR [3, 4], we now provide a concise, descriptive addendum that focuses on how the projection is derived, executed, and documented during balloon‐expandable TAVR. It contributes to the field of TAVR by extending fluoroscopic concepts used to visualize coronary ostia and bifurcations in coronary interventions [5].

In routine practice, THV deployment is often performed in 3C and/or CO projections, yet the LM ostium is frequently not visible during implantation. The LM‐View fluoroscopic projection brings the LM ostium into view at the time of deployment, enhancing spatial awareness of the THV stent‐frame outflow (neo‐skirt plane) relative to the LM ostium (coronary risk plane). This anatomic relationship frequently influences redo‐TAVR feasibility in patients who outlive their index valve.

The LM‐View projection is a patient‐specific fluoroscopic orientation that is straightforward to derive from pre‐procedural computed tomography (CT). After alignment of the annular plane, the right and non‐coronary cusps (RCC/NCC) are overlapped along the annular S‐curve to isolate the LM ostial take‐off. In practice, this most often results in a left anterior oblique (LAO) with cranial angulation. Importantly, the LM‐View is not intended to replace established three‐cusp (3C; typically LAO‐caudal) or cusp‐overlap (CO; typically right anterior oblique‐caudal) views, which remain the standard fluoroscopic references for balloon‐expandable THV implantation. Rather, the LM‐View is proposed as a complementary projection that may enhance spatial awareness of the LM ostium relative to the THV stent‐frame outflow during implantation, particularly in the context of lifetime management and redo‐TAVR planning. The current data primarily support feasibility, with validation in larger cohorts necessary to assess potential clinical impact.

Video‐1 shows the workflow: In the catheterization laboratory, operators first confirm LM visualization with a brief root angiogram in the CT‐derived LM‐View, then neutralize still crimped yet already positioned in the aortic annulus THV stent‐frame parallax, if present. Depth control is standardized by positioning the balloon mid‐marker at the bottom of the NCC while keeping the LM ostium in view throughout deployment. After inflation, a short, parallax‐free “corrected” LM‐View angiogram is acquired for assessment of coronary risk/neo‐skirt planes and archiving. When extreme angles are impractical, other fluoroscopic projections should be used. Documentation is brief and reproducible: in the “corrected” LM‐View, the neo‐skirt is classified as below the LM ostium nadir, at the LM (between nadir/zenith), or above the LM ostium zenith. This documentation enables meaningful cross‐case comparison and provides a pragmatic on‐table screening of redo feasibility, with definitive planning reserved for CT [6].

In conclusion, this focused description emphasizes the practical steps that make the LM‐View projection easy to implement and straightforward to report, facilitating alignment of the index THV relative to the LM ostium in selected patients with the aim of enhancing redo‐TAVR feasibility. This projection may be used either as an independent fluoroscopic reference during index‐TAVR or in combination with established projections as a complementary view, and may be particularly useful during valve‐in‐valve procedures, in which THV positioning is referenced to the first implanted valve, and the LM‐View isolates the true LM ostium, which is often the procedural focus. The current data primarily support feasibility, and validation in larger cohorts will be required to determine potential clinical impact.

## Disclosure

J.W. is a consultant for Edwards Lifesciences and receives research funding from Edwards Lifesciences, Medtronic, and Boston Scientific. J.J. does not have any relevant disclosures.

## Conflicts of Interest

The authors declare no conflicts of interest.

## Supporting information


**Video‐1:** THV deployment in LM‐View. LM – left main. LCC – left coronary cusp. NCC – non‐coronary cusp. RCC – right coronary cusp.
